# Ontario Veterinary College

**Published:** 1899-06

**Authors:** 


GRAND RAPIDS VETERINARY COLLEGE.

---

### AMONG THE COLLEGES.

### ONTARIO VETERINARY COLLEGE.

*Class Standing.*

Disease and Treatment: Seniors—W. J. R. Fowler, first prize; J. Corrigan, second prize; J. F. Olweiler and William L. Rundle, third prize, equal. Honors—V. J. Andre, H. W. Carley-Baker, John Black, F. B. Davidson, J. J. Giblin, W. M. Goff, W. J. Hennessey, A. Hobbs, D. A. Irvine, George Jerome, J. C. Jones, L. A. Judson, H. F. Kaufman, Joseph King, C. Manning, F. Neff, H. J. Pugsley, W. S. Richards, J. L. Shorey, J. E. Sommer, W. A. Sproule, J. M. Young.

Materia Medica: Seniors—W. J. R. Fowler, first prize; F. B. Davidson, second prize; F. C. Jones, third prize. Honors—H. W. Carley Baker, J. W. Corrigan, Wesley M. Goff, W. J. Hennessey, D. A. Irvine, H. F. Kaufman, Joseph King, C. Manning, J. F. Olweiler, H. Pugsley, H. Ramsey, W. S. Richards, W. L. Rundle, J. L. Shorey, James M. Young.

Chemistry: Seniors—W. J. R. Fowler, first prize; W. M. Goff, second prize; J. M. Young, third prize. Honors—V. J. Andre, H. W. Carley-Baker, J. H. Black, J. W. Corrigan, F. B. Davidson, G. Jerome, P. H. Morin, N. L. Richards, H. O. Ramsey, W. A. Sproule.

Pathology: Seniors—W. J. R. Fowler, first prize; J. M. Young, second prize. Honors—H. W. Carley-Baker, J. Black, J. Corrigan, P. B. Davidson, William Goff, W. J. Hennessey, D. A. Irvine, F. C. Jones, G. Jerome, H. F. Kaufman, Joseph King, J. McVicar, C. Manning, P. M. Morin, H. J. Pugsley, W. S. Richards, W. L. Rundle, J. Shorey, W. A. Sproule.

Physiology: Seniors—W. J. R. Fowler, first prize; H. W. Carley-Baker, second prize; John W. Corrigan, third prize. Honors—F. B. Davidson, C. J. Donnelley, Westley M. Goff, D. A. Irvine, George Jerome, F. C. Jones, H. F. Kaufman, Joseph King, C. A. Locke, C. L. Manning, J. F. Olweiler, H. J. Pugsley, W. S. Richards, William L. Rundle, J. L. Shorey, W. A. Sproule, E. M. Saigeon, J. M. Young.

Anatomy : Seniors—W. J. R. Fowler, first prize, silver medal; J. M. Young, second prize; J. F. Olweiler, third prize. Honors—V. J. Andre, H. W. Carley-Baker, J. Corrigan, F. B. Davidson, W. M. Goff, G. Jerome, F. C. Jones, Joseph King, H. F. Kaufman, T. J. Kirwan, C. A. Locke, C. L. Manning, J. M. Mitchell, J. McDougall, H. J. Pugsley, A. J. Roll, W. L. Rundle, H. O. Ramsey, W. A. Sproule, J. L. Shorey.

Entoyzoa : Seniors—J. W. Corrigan, prize. Honors—H. W. Carley-Baker, T. B. Davidson, W. J. R. Fowler, W. M. Goff, F. C. Jones, D. A. Irvine, F. J. Neff, George Jerome, Joseph King, H. F. Kaufman, A. H. Morin, J. F. Olweiler, W. L. Rundle, W. S. Richards, H. O. Ramsey, Ed. M. Saigeon, J. L. Shorey, W. L. Schultze, James M. Young.

Dissected Specimens : Seniors—Victor J. Andre, gold medal, given by Toronto Industrial Exhibition Association; T. J. Kirwan, second prize.

Best general examination : W. J. R. Fowler, gold medal, given by the Ontario Veterinary Association. Honors—J. W. Corrigan, T. B. Davidson, Joseph King.

Primary Examination : Anatomy—Alva G. King, J. Chris. Osborne, M. Philps, D. J. Smith. Materia Medica—H. James Elliott, Arthur N. Norwood, D. J. Smith, Jacob Van Hunt.

Disease and Treatment : Juniors—R. M. Nyblett, first prize; J. B. Foster, second prize; W. K. Lewis and C. D. McGillivray, equal, third prize. F. Aldous, J. Campbell, J. Dellenberger, H. J. Daniels, D. L. DeWolf, D. K. Dow, F. Erwin, R. Frame, J. Freeman, H. J. Hagerty, O. Hartnage, L. Jargo, W. H. Jordan, E. E. Linton, J. Lucas, J. McFadyean, J. D. McLeod, A. C. Ramsay, J. Rennie, G. A. Shaw, T. M. Shippee, E. J. Shirley, G. O. Smith, A. T. Smith, T. A. White. Students entering January 1, 1899—L. M. Holmes, H. Killip, O. J. Phillips, I. B. Rivenburgh.

Anatomy : Juniors—C. D. McGilvray, first prize; W. K. Lewis, second prize; J. P. Foster, third prize. Honors—D. S. DeWolf, B. K. Dow, F. Erwin, H. J. Hagerty, L. Jurgo, W. H. Jordan, D. S. Krull, E. S. Linton, James Lucas, R. M. Nyblett, A. C. Ramsay, A. F. Smith, Thomas Shipper.

Physiology : Juniors—J. P. Foster, first prize; R. M. Nyblett, second prize; C. D. McGilvray, third prize. Honors—J. A Campbell, B. K. Dow, D. S. DeWolf, F. E. Erwin, R. Frame, J. Freeman, — Hartnage, L. M. Holmes, W. H. Jordan, L. Jargo, E. Linton, James Lucas, W. K. Lewis, J. McFaydean, A. C. Ramsay, T. Shippee, A. F. Smith, G. O. Smith.

Chemistry: Juniors—C. D. McGilvray, first prize; J. P. Foster, second prize; R. M. Nyblett, third prize. Honors—R. Frame, W. H. Jordan, L. Jargo, W. K. Lewis, J. McFaydean, G. A. Shaw, G. O. Smith.

Pathology: Juniors—C. D. McGilvray, first prize; A. F. Smith, second prize; J. P. Foster, third prize. Honors—T. Campbell, — DeWolf, B. K. Dow, Robert Frame, T. M. Holmes, H. J. Hagerty, L. Jargo, George Jermyn, W. Jordan, C. Linton, J. Lucas, W. K. Lewis, R. M. Nyblett, A. C. Ramsay, George Smith, J. Shirley, S. White.

---

No less than six graduates of other veterinary schools added the degree of the McKillip Veterinary College to their previous attainment at other schools. The large clinical opportunities at the McKillip College make it very attractive for those desiring more extended studies or a post-graduate course in clinical work.

